# Site-specific structural order in Alzheimer's Aβ42 fibrils

**DOI:** 10.1098/rsos.180166

**Published:** 2018-07-04

**Authors:** Hongsu Wang, Yoon Kyung Lee, Christine Xue, Zhefeng Guo

**Affiliations:** Department of Neurology, Brain Research Institute, Molecular Biology Institute, University of California, Los Angeles, CA 90095, USA

**Keywords:** amyloid-β, EPR, protein aggregation, spin labelling, Alzheimer's disease, neurodegenerative disease

## Abstract

Deposition of amyloid fibrils is a pathological hallmark of Alzheimer's disease. Aβ42 is the major protein whose aggregation leads to the formation of these fibrils. Understanding the detailed structure of Aβ42 fibrils is of particular importance for delineating the mechanism of Aβ42 aggregation and developing specific amyloid-targeting drugs. Here, we use site-directed spin labelling and electron paramagnetic resonance spectroscopy to study the site-specific structural order at each and every residue position in Aβ42 fibrils. Strong interactions between spin labels indicate highly ordered protein backbone at the labelling site, while weak interactions suggest disordered local structure. Our results show that Aβ42 consists of five β-strands (residues 2–7, 10–13, 17–20, 31–36, 39–41), three turns (residues 7–8, 14–16, 37–38) and one ordered loop (residues 21–30). Spin labels introduced at β-strand sites show strong spin–spin interactions, while spin labels at turn or loop sites show weak interactions. However, residues 24, 25 and 28 also show strong interactions between spin labels, suggesting that the loop 21–30 is partly ordered. In the context of recent structural work using solid-state NMR and cryoEM, the site-specific structural order revealed in this study provides a different perspective on backbone and side chain dynamics of Aβ42 fibrils.

## 1. Introduction

Deposition of amyloid-β (Aβ) fibrils in the form of amyloid plaques is a pathological hallmark of Alzheimer's disease [1]. The protein Aβ is a fragment of a transmembrane protein called amyloid precursor protein, resulting from proteolytic digestion by β- and γ-secretases [2]. There are two major species of Aβ: the 40-residue Aβ40 and the 42-residue Aβ42. The only difference between Aβ40 and Aβ42 is that Aβ42 has two extra residues at the C-terminal end. Although Aβ40 is severalfold more abundant than Aβ42, Aβ42 is the major, and sometimes only, component of amyloid plaques in the brain [3–6]. Aβ40 and Aβ42 have been shown to interact with each other during aggregation [7,8] and they form interlaced fibrils *in vitro* [9], supporting the observation that the Aβ42/Aβ40 ratio is a better predictor of Alzheimer's risk than the Aβ42 concentration alone [10–12]. Extensive research effort has been devoted to studying the structure of Aβ42 fibrils, and has led to some breakthrough findings in the past few years. Solid-state NMR studies by Xiao *et al*. [13], Wälti *et al*. [14] and Colvin *et al*. [15] have provided atomic details on Aβ42 fibril structure. Schmidt *et al*. [16] reported a 7-Å structural model of Aβ42 fibrils based on cryoEM data. More recently, Gremer *et al*. [17] used a combination of cryoEM and solid-state NMR to determine the structure of Aβ42 fibrils at 4-Å resolution. However, structural differences still exist among these models from different studies, likely reflecting the polymorphic nature of Aβ42 fibrils. Further structural investigations are needed to explore the diversity of Aβ42 fibril structures.

Here, we report a structural study of Aβ42 fibrils using site-directed spin labelling and electron paramagnetic resonance (EPR) spectroscopy. The general strategy of site-directed spin labelling is to first introduce a cysteine residue at a position of interest using site-directed mutagenesis, and then the cysteine residue is modified with a spin-labelling reagent. In this work, a commonly used spin label called R1 is used. The spin-labelled Aβ42 proteins were then used to prepare amyloid fibrils, which were studied with EPR. Because the Aβ42 fibrils adopt a parallel in-register structure [18–20], the spin label side chains form a ladder, stacking on top of each other. The stacking of spin labels leads to a strong spin exchange interaction between spin labels, which in turn gives rise to a characteristic single-line EPR lineshape [8,18]. We have previously shown that the quantitative analysis of the spin exchange interaction can be used to determine the location of β-strands, turns and loops [21]. The rationale is that strong spin exchange interactions between spin labels require structural rigidity of the protein backbone, which is provided by the β-sheet structure in the fibrils, but not provided by loosely packed turns or loops. Therefore, the EPR spectrum of the spin-labelled amyloid fibrils represents the structural order at the labelling position.

In this work, we have obtained a full set of EPR spectra of Aβ42 fibrils covering every residue position of the Aβ42 sequence. Using spectral simulations, we were able to quantify the strength of spin exchange interaction as a measure of the structural order at each and every residue position in Aβ42 fibrils. The results show that Aβ42 consists of five β-strands (2–7, 10–13, 17–20, 31–36, 39–41), three turns (7–8, 14–16, 37–38) and one ordered loop (21–30). The relevance of our findings in the context of other structural studies is discussed.

## 2. Results and discussion

We have previously shown that EPR spectra of spin-labelled amyloid fibrils can be used to obtain secondary structure information [8,21]. The basis of this analysis is the spin exchange interaction between spin labels in a parallel in-register β-sheet structure, which has been found in the amyloid fibrils of most proteins studied to date [22]. In a parallel in-register β-sheet structure, the side chains of the same residue position, but from different Aβ42 proteins, are stacked on top of each other, forming a ladder. When a spin label is introduced, the stacking of the spin label side chain results in the exchange of the unpaired electrons located in the nitroxide group of the spin label. The spin exchange interaction leads to the collapse of the three resonance lines to a single-line spectrum. The strength of the spin exchange interaction depends on the backbone structure of the labelling site. Spin labels located on β-strands give strong spin exchange interactions, while spin labels located on turns or disordered loops give weak spin exchange interaction. Therefore, the quantitative analysis of the spin exchange interaction using spectral simulations can be used to determine the secondary structure in amyloid fibrils.

**Figure 1.**
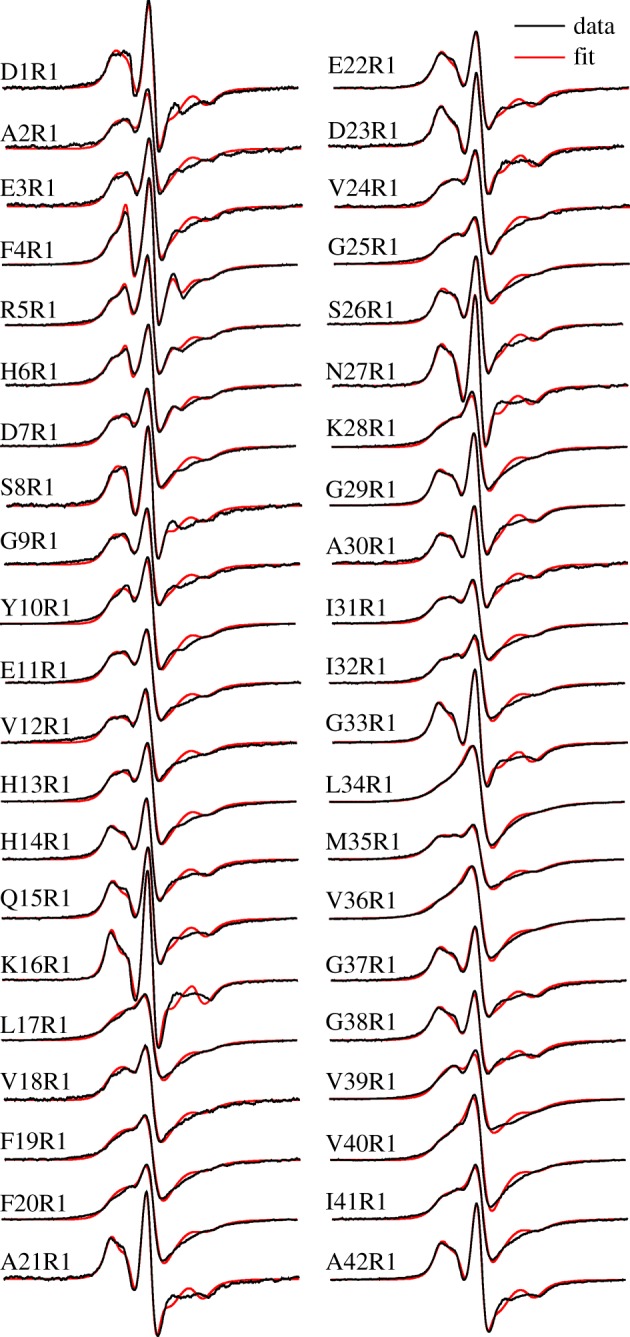
EPR spectra of Aβ42 fibrils with spin labels introduced at indicated residue positions. The spin label is named R1. Experimental spectra are shown in black, and best fits from spectral simulations are shown in red. Note that the EPR spectra with strong spin exchange interactions are characterized by the single-line feature, such as L34R1 and V36R1, while the EPR spectra with weak spin exchange interactions are characterized by three resonance lines, such as K16R1 and A42R1. All spectra are normalized to the same number of spins. The scan width is 200 G.

**Figure 2.**
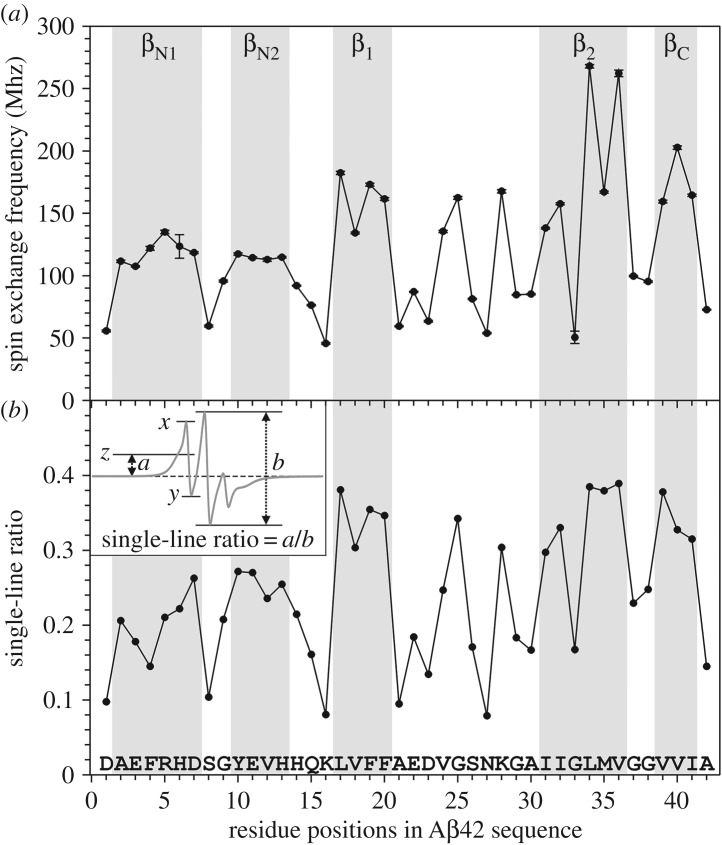
Quantitative analysis of spin exchange interactions reveals site-specific structural order in Aβ42 fibrils. (*a*) Plot of spin exchange frequencies, obtained from spectral simulations, as a function of residue positions. The β-strands are assigned to consecutive residue positions with high spin exchange frequencies. (*b*) Plot of single-line ratio as a function of residue positions. The inset shows how the single-line ratio is determined. In the inset, line *z* is at the mid-point between *x* and *y*. For EPR spectra without spin exchange interactions, line *z* would be at the same level as the baseline, and the single-line ratio would be zero. For EPR spectra with strong spin exchange interactions, *x* and *y* would converge to a single inflection point. For the strongest spin exchange interactions, the low-field feature to the left of the centre peak is completely smoothed out, and the single-line ratio would be arbitrarily set at 0.5.

Previously we have obtained 22 EPR spectra of Aβ42 fibrils, and each sample was spin-labelled at a different residue position [8]. Here, we completed the spin label scanning of the full Aβ42 sequence, and are reporting all 42 EPR spectra of the spin-labelled Aβ42 fibrils (figure 1). The increased spatial resolution allows us to obtain residue-specific structural information in Aβ42 fibrils. Spin exchange frequency was obtained by fitting the experimental EPR data to simulated spectra (figure 1). The spin exchange frequency as a function of residue position is plotted in figure 2*a*. We also assessed the strength of the spin exchange interaction using an empirical parameter, the single-line ratio, which we introduced in a previous analysis of spin-labelled yeast prion Ure2 fibrils [21]. The calculation of single ratio is slightly modified to better reflect the strength of spin exchange interactions (figure 2*b*). The advantage of the single-line ratio is that it does not require spectral simulation, thus it is immune to any errors associated with spectral simulation and fitting. The disadvantage of the single-line ratio is that it is insensitive to changes in spin exchange interactions when the interactions are strong. Importantly, both the spectral simulations (figure 2*a*) and single-line ratio (figure 2*b*) gave very similar results regarding the strength of spin exchange interactions in Aβ42 fibrils. Based on EPR data, we identified five β-strands (2–7, 10–13, 17–20, 31–36, 39–41), three turns (7–8, 14–16, 37–38) and one ordered loop (21–30). The spin exchange interactions are stronger for β-strand sites than those residue positions in turns or loops. We also identified three positions (residue 24, 25, 28) in the loop 21–30 that gave strong exchange interactions, suggesting that this loop is also partly ordered.

In the last few years, significant progress has revealed atomic details of the structures of Aβ42 fibrils. These advances came from studies using solid-state NMR [13–15] and cryoEM [16,17]. Structural models from these studies have similarities, and also some differences. Some of these structural differences may result from different fibril preparation conditions. The fibrils of Gremer *et al*. [17] were prepared in an acidic buffer containing organic solvent (30% acetonitrile and 0.1% trifluoroacetic acid). The fibrils of Wälti *et al*. [14] were prepared in a pH 7.4 buffer at 37°C with agitation, and repeated seeding was used to obtain homogeneous Aβ42 fibrils. The fibrils of Colvin *et al*. [15] were prepared with seeds in a pH 8.0 buffer at room temperature without agitation, and the Aβ42 sequence contains an extra methionine residue at the N-terminus. The fibrils of Xiao *et al*. [13] were prepared in a pH 7.4 buffer at room temperature with slow rotation. The different fibril preparation conditions may lead to the formation of different fibril polymorphs. Detailed chemical shift comparisons by Wälti *et al*. [14] and Colvin *et al*. [15] suggest that their structural models of Aβ42 fibrils are essentially the same as the one reported by Xiao *et al*. [13]. Further comparison of the models by Wälti *et al*. [14] and Colvin *et al*. [15] shows that these two models are of the same polymorph [23]. The structure of Gremer *et al*. [17] is of another polymorph due to vastly different fibril preparation conditions.

The Aβ42 fibrils studied in this work were prepared in a phosphate buffer (pH 7.4) at room temperature with agitation, similar to the condition used by Xiao *et al*. [13]. Because the fibrils of Wälti *et al*. [14] and Colvin *et al*. [15] were prepared without agitation, and the same fibril polymorph was obtained from these studies, it appears that agitation did not lead to the formation of other polymorphs. This is different from the case of Aβ40 fibrils, which form different polymorphs under quiescent and agitated conditions [24,25]. Fibril polymorphism may pose a potential complication for the analysis of the EPR data. Because spin labels were introduced for EPR studies, it is possible that spin labelling could cause the formation of a different fibril polymorph. Ideally, this should be investigated with another technique that has sufficient structural resolution to distinguish different fibril polymorphs. In the case of Aβ40 fibrils, quiescent and agitated conditions produce fibrils with different morphologies as revealed by transmission electron microscopy [18,24,25]. We have previously shown that spin-labelled Aβ42 fibrils have similar morphology as the wild-type fibrils [8]. However, similar morphologies between the fibrils of Gremer *et al*. [17] and the fibrils of Xiao *et al*. [13], Wälti *et al*. [14] and Colvin *et al*. [15] suggest that transmission electron microscopy may not have the distinguishing power for Aβ42 fibril polymorphism. Our approach for this potential complication is to emphasize the overall trend of spin exchange interactions among adjacent labelling sites, rather than the absolute value of spin exchange frequency at a particular site. One example of this strategy is the G33R1 mutant. The EPR spectrum of G33R1 (figure 1) and the quantitative analysis of spin exchange interactions (figure 2) show weak spin exchange interactions. This might be because G33R1 formed fibrils of a different structure in which residue 33 is located at a disordered region. But, its neighbouring residues on both sides (residues 32 and 34) have strong spin exchange interactions (figure 2). Therefore, we interpret site 33 as part of a β-strand that runs from residues 31 to 36. This issue is similarly addressed in our Rosetta modelling, during which the secondary structure information from EPR was used in combination of full-atom Rosetta energy functions [8], and the final structure was not simply dictated by EPR data.

The EPR spectra of spin-labelled Aβ42 fibrils contain information on the site-specific structural order at each of the 42 residue positions (figure 1). Using quantitative analysis of the spin exchange interactions, we found that Aβ42 fibrils adopt five β-strand regions (figure 2). Two of the β-strand regions include residues 17–20 and 31–36, and we named these two β-strands β_1_ and β_2_. We think that these two β-strands correspond to similar β-strand regions in Aβ40 [19,20,26], and they may be the structural basis for the cross-seeding phenomenon between Aβ40 and Aβ42 [7,8], and for the formation of interlaced Aβ40–Aβ42 fibrils [9]. One β-strand (β_C_), consisting of residues 39–41, is located at the C-terminal end, and this β-strand is the distinguishing feature of Aβ42 fibrils. Owing to the shorter length of the Aβ40 sequence, Aβ40 is incapable of forming this C-terminal strand. Our EPR data also reveal two β-strands (β_N1_ for residues 2–7 and β_N2_ for residues 10–13) in the N-terminal region (figure 2), although these two β-strands have weaker spin exchange interactions than β_1_, β_2_ and β_C_, suggesting the N-terminal β-strands are less ordered than other β-strands. In solid-state NMR studies, the first 10 residues are missing in the fibril models of Xiao *et al*. [13] and Colvin *et al*. [15]. In the solid-state NMR studies of Wälti *et al*. [14], the first 14 residues are partly ordered. In the cryoEM studies of Schmidt *et al*. [16], the resolved electron density for the N-terminal region is much weaker than for the rest of the sequence. The recent work by Gremer *et al*. [17] combined cryoEM and solid-state NMR to determine the structure of Aβ42 fibrils and has resolved electron density for all the N-terminal residues. Gremer *et al*. [17] showed that N-terminal residues participate in a hydrophobic cluster consisting of residues A2, F4, L34 and V36. In our Rosetta model, the N-terminal region is stabilized by electrostatic interactions among residues E3, R5, D7, E11 and H13. Owing to the lower structural order of β_N1_ and β_N2_ than that of other β-strands, the N-terminal β-strands likely play a less critical role in the structural stability of Aβ42 fibrils. Based on our Rosetta model and the lack of contacts between the N-terminal region and the rest of the fibril core from solid-state NMR studies [13–15], we speculate that the N-terminal strands may modulate the packing between Aβ42 protofilaments.

**Figure 3.**
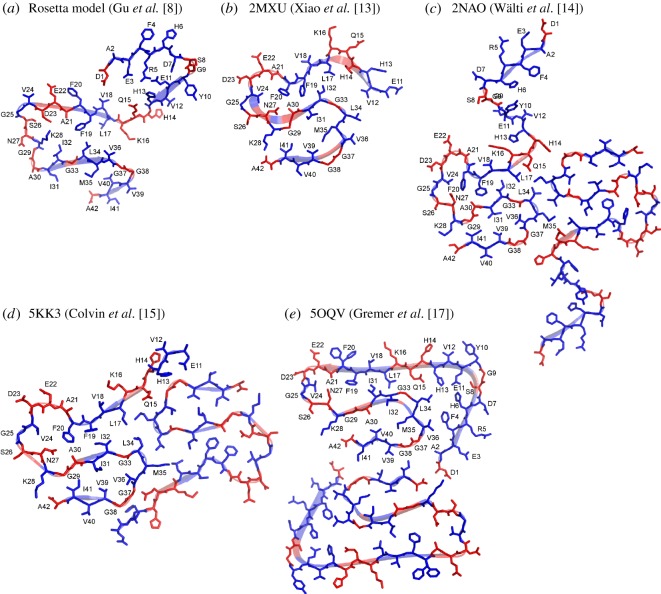
Comparison of Aβ42 fibril models in the context of site-specific structural order as determined from EPR data. The structural model based on EPR data and Rosetta modelling is shown in panel (*a*). Four recent structural models of Aβ42 fibrils from the Protein Data Bank are shown in panels (*b*)–(*e*). The secondary structure is shown as ribbons, and the information on the secondary structure is taken directly from PDB files. Residues with strong spin exchange interactions are shown in blue, and residues with weak spin exchange interactions are shown in red.

In comparison with other Aβ42 fibril structures, we graphed the Rosetta model that we previously reported in Gu *et al*. [8], together with four recent structural models deposited in the Protein Data Bank (PDB): 2MXU by Xiao *et al*. [13], 2NAO by Wälti *et al*. [14], 5KK3 by Colvin *et al*. [15] and 5OQV by Gremer *et al*. [17] (figure 3). In the graph, β-strands are shown as ribbons as specified in their PDB files. Residues with strong spin exchange interactions are coloured in blue, while residues with weak spin exchange interactions are coloured in red. A common feature among all these structural models is that residues 17–42 adopt an S-shaped structure. There are two turns in this S-shaped structure. The first turn is located at residues 22–28, and the second turn is near residues 37–38. The N-terminal region is less defined in all models except Gremer *et al*. [17], which shows extensive contact between the C-terminal residues and the rest of the structure. Notably, there are still considerable amounts of structural differences between these structures. All the structures solved by solid-state NMR and cryoEM adopt an S-shaped fold, but detailed side chain packing differs. For example, the side chains of F19 and F20 point to the interior of the fibril core in Xiao *et al*. [13], Wälti *et al*. [14] and Colvin *et al*. [15], but point to the opposite sides of a β-strand in Gremer *et al*. [17] with only F19 pointing to the fibril core. Interestingly, F19 and F20 are also pointing to opposite sides of a β-strand in Aβ40 fibril structures [19,20,26]. The significance of these subtle structural differences is not immediately clear. To gain a complete understanding of Aβ42 fibril structure and Aβ aggregation, future studies are needed to reveal the structural differences between different structural polymorphs, and to understand how different structural polymorphs may play distinct roles in the pathogenesis of Alzheimer's disease.

## 3. Material and methods

### 3.1. Preparation of Aβ42 proteins and spin labelling

The plasmids of wild-type GroES-ubiquitin-Aβ42 [27] and the deubiquitylating enzyme Usp2-cc [28] were kindly provided by Dr. Il-Seon Park at Chosun University (South Korea) and Dr. Rohan T. Baker at Australian National University (Australia). Cysteine mutations were introduced using the QuikChange method (Agilent) and the DNA sequence was confirmed with sequencing.

The cysteine mutants of Aβ42 were expressed in *Escherichia coli* and purified with a nickel column as previously described [8,29]. Full-length Aβ was then cleaved from the fusion protein with Usp2-cc as described [30]. For spin labelling, the spin labelling reagent MTSSL (1-oxyl-2,2,5,5-tetramethylpyrroline-3-methyl methanethiosulfonate, AdipoGen Life Sciences) was used and detailed protocols have been previously described [8,30]. The spin-labelled Aβ42 proteins were lyophilized and stored at −80°C.

### 3.2. Preparation of Aβ42 fibrils

Lyophilized Aβ42 powder was first dissolved in 100% 1,1,1,3,3,3 hexafluoro-2-propanol (HFIP) to 1 mM final Aβ concentration, and was bath sonicated for 5 min and incubated at room temperature for 30 min. HFIP was evaporated overnight in the fume hood and the Aβ samples were then put under vacuum for 1 h. To prepare fibrils, HFIP-treated Aβ was dissolved in a CG buffer (20 mM CAPS, 7 M guanidine hydrochloride, pH 11) to 1 mM concentration, then was diluted 20-fold to a PBS buffer (50 mM phosphate, 140 mM NaCl, pH 7.4) so that the final Aβ concentration was 50 µM. Then Aβ samples were placed on a digital vortex mixer with a shaking speed of 600 rpm at room temperature. Fibril growth was monitored daily with thioflavin T fluorescence. After thioflavin T fluorescence reached a plateau (5–7 days), fibrils were collected by centrifugation at 14 000 *g* for 20 min. The fibril pellet was washed twice with the PBS buffer.

### 3.3. Electron paramagnetic resonance spectroscopy and spectral simulations

For EPR, the fibrils were resuspended in approximately 20 µl of the PBS buffer. The fibril sample was then loaded into glass capillaries (VitroCom) sealed at one end. A modulation frequency of 100 kHz was used. EPR measurements were performed at 20 mW microwave power at room temperature on a Bruker EMX EPR spectrometer. Modulation amplitude was optimized for individual spectra (typically 4 G). Typically 10 to 30 scans were averaged for each EPR spectrum. Spectral simulations were performed using the program MultiComponent, written by Dr. Christian Altenbach at University of California, Los Angeles. A microscopic order macroscopic disorder model was used [31]. A least-squares fit of the user-defined spectral parameters was performed using the Levenberg–Marquardt algorithm. Among the previously published 22 EPR spectra of spin-labelled Aβ42 fibrils [8], the spectra of G29R1, L34R1, G37R1 and A42R1 were obtained using the fibrils prepared in this work. Additional 20 EPR spectra were obtained to complete the full set of 42 residue positions. Detailed fitting procedure and fitting parameters have been previously described [8].

## Data accessibility

Data are available from the Dryad Digital Repository: http://dx.doi.org/10.5061/dryad.768dk4s [32].
